# Accumulation of health risk behaviours is associated with lower socioeconomic status and women's urban residence: a multilevel analysis in Japan

**DOI:** 10.1186/1471-2458-5-53

**Published:** 2005-05-27

**Authors:** Yoshiharu Fukuda, Keiko Nakamura, Takehito Takano

**Affiliations:** 1 Health Promotion/International Health, Division of Public Health, Graduate School of Tokyo Medical and Dental University, 1-5-45 Yushima, Bunkyo-ku, Tokyo 113-8519, Japan

## Abstract

**Background:**

Little is known about the socioeconomic differences in health-related behaviours in Japan. The present study was performed to elucidate the effects of individual and regional socioeconomic factors on selected health risk behaviours among Japanese adults, with a particular focus on regional variations.

**Methods:**

In a nationally representative sample aged 25 to 59 years old (20,030 men and 21,076 women), the relationships between six risk behaviours (*i.e.*, current smoking, excessive alcohol consumption, poor dietary habits, physical inactivity, stress and non-attendance of health check-ups), individual characteristics (*i.e.*, age, marital status, occupation and household income) and regional (N = 60) indicators (per capita income and unemployment rate) were examined by multilevel analysis.

**Results:**

Divorce, employment in women, lower occupational class and lower household income were generally associated with a higher likelihood of risk behaviour. The degrees of regional variation in risk behaviour and the influence of regional indicators were greater in women than in men: higher per capita income was significantly associated with current smoking, excessive alcohol consumption, stress and non-attendance of health check-ups in women.

**Conclusion:**

Individual lower socioeconomic status was a substantial predictor of risk behaviour in both sexes, while a marked regional influence was observed only in women. The accumulation of risk behaviours in individuals with lower socioeconomic status and in women in areas with higher income, reflecting an urban context, may contribute to their higher mortality rates.

## Background

Health inequality within the population is a major public health concern [1,2]. Individual socioeconomic status, as measured by educational level, occupational class and income, has been shown to be closely related to both mortality and morbidity in combination with race/ethnicity, access to health care services and a number of other factors [3,4]. Previous studies confirmed by that the relationship between socioeconomic factors and health status can be explained in part by differences in health-related behaviour according to socioeconomic status [5,6]. Generally, people with lower socioeconomic status have a higher likelihood of exposure to risk behaviours, such as smoking, excessive alcohol consumption, physical inactivity, poor diet and non-attendance of health check-ups, as well as psychological stress [7-13].

A number of recent studies, especially a series of multilevel analyses, have focused on the regional and contextual effects on health [14-16], and have demonstrated effects on mortality, morbidity and self-rated health [17-20]. Regional socioeconomic conditions have been shown to influence health-related behaviour independently and interactively with individual socioeconomic status, and most previous studies indicated a higher likelihood of health risk behaviour in people living in socioeconomically disadvantaged areas [21-23].

Japan has the longest life expectancy and healthy life expectancy in the world [24], which has been shown to be due not only to improvements in the standard of living due to economic growth, but also to the relatively small degree of socioeconomic disparity within the population [25,26]. We are especially interested in the degree of socioeconomic inequalities in health and its relation to the status of the Japanese population as the healthiest worldwide, as well as the future implications.

There is only limited evidence of socioeconomic inequality in health in Japan. National data indicate a substantial gap in mortality among occupations: lower occupational classes, such as service workers and primary industry workers, show higher mortality rates than higher occupational classes, such as professionals and technical workers [27]. Previous studies demonstrated regional variations in mortality among municipalities, and areas with lower socioeconomic status were associated with higher rates of mortality [28]. However, recent figures have indicated clear gender differences in the relationships between area socioeconomic status and mortality: areas with higher income and educational level show lower mortality rates among men, but not among women [28].

Although a few studies in Japan have demonstrated a significant correlation between lower socioeconomic status and increased likelihood of risk behaviour at the individual level, the relationship was moderate as compared to that in other industrial nations [29,30]. At the regional level, there are substantial variations in the prefectural prevalence of smoking, alcohol drinking and other health risk behaviours [31]. Nevertheless, little is known about the independent impacts of individual and regional socioeconomic conditions on health risk behaviour, paying attention to integration of the influences at both levels in Japan.

The present study was performed to examine the relationships of individual and regional socioeconomic factors to health risk behaviour with regard to smoking, alcohol consumption, diet, exercise, psychological stress and attendance at health check-ups, by multilevel analysis of a nationally representative sample in Japan.

## Methods

### Data source

The 2001 Comprehensive Survey of the Living Conditions of People on Health and Welfare conducted by the Ministry of Health, Labour and Welfare [32] was used for analysis in the present study. All members of households within 5,240 area units, sampled randomly with stratification by prefecture in Japan, were interviewed. The survey included household and individual basic information regarding demographics, health, illness profiles, lifestyle and other items. The total number of households sampled for basic information was 247,195, of which 30,386 were interviewed with regard to income and savings. The response rates were 87.4% for the basic information survey and 79.5% for the income survey. Microdata files from this survey were used with permission from the Ministry of Public Management, Home Affairs, Posts and Telecommunications; we analysed the data for 20,030 men and 21,076 women aged 25 to 59 years whose basic and income data were surveyed.

### Health risk behaviour

The following six health risk behaviours were used in the analyses: current smoking, excessive alcohol consumption, poor dietary habits, physical inactivity, stress and non-attendance of health check-ups.

#### Current smoking

Smoking habits were surveyed based on the following four categories: (a) "I don't smoke"; (b) "I smoke every day"; (c) "I smoke on occasion but not every day"; and (d) "I have stopped smoking for more than one month". We categorised (b) and (c) as current smokers.

#### Alcohol consumption

Alcohol consumption per day was surveyed, and excessive alcohol consumption was defined as more than 2.0 "gou" per day (one "gou" is a measure of 180 ml of Japanese sake, and contains almost 20 g of ethanol).

#### Poor dietary habits

Dietary habits were surveyed using the question, "What are your daily dietary practices?": have regular meals; have balanced meals; have bland (less salty) meals; and do not overeat. People who answered "no" to all these questions were defined as having poor dietary habits.

#### Physical inactivity

The question was, "Do you exercise or play sports regularly?" People who answered "no" were defined as being physically inactive.

#### Stress

The question was, "Do you have any stress or worries in your daily life?" People who answered "yes" were defined as being stressed.

#### Non-attendance of health check-ups

The subjects' attendance of health check-ups in the past year was surveyed. Health check-ups included all types of general health check-up, such as periodical health examinations in the workplace, health examinations in communities and multiphasic examinations ("Ningen Dock"), but not health examinations only for cancer screening, maternal health check-ups, dental health examinations or clinical examinations at medical facilities.

### Individual socioeconomic variable

We used age, marital status, occupation and income as individual socioeconomic variables.

Subjects' marital status was categorised as married, single, widowed or divorced. Occupation classification was based on the Vital Statistics in Japan [27]: professional and technical workers (professional); managers and officials (manager); clerical and related workers (clerk); sales workers (sales work); service workers (service work); protective service workers (protective service); agriculture, forestry and fishery workers (agriculture); workers in transport and communications (transport); craftsmen, mining, production process and construction workers and labourers (labour); housework; and others including workers not classifiable by occupation, the unemployed and students.

Annual household income before tax, including benefits and inheritance, was used as income information. To adjust for family size and composition, we applied the modified OECD equivalence scale of 1.0 for the first adult, 0.5 for the second and each subsequent person aged 14 and over, and 0.3 for each child under 14 [33]. The subjects were divided into quintiles according to equivalent income, and the income quintile was used as an independent variable.

### Regional socioeconomic variables

Japan consists of 47 prefectures, and the prefecture was used the basic regional unit in the present study. For prefectures including a metropolitan city/cities, *i.e.*, the Tokyo metropolitan area (23 special wards, "ku") and 12 cities designated by ordinance (Sapporo, Sendai, Chiba, Yokohama, Kawasaki, Nagoya, Kyoto, Osaka, Kobe, Hiroshima, Kitakyushu and Fukuoka), the regional unit was divided into prefectures excluding metropolitan cities. Consequently, the individual data were linked with 47 prefectures and 13 metropolitan cities.

Per capita income and unemployment rate of prefectures and metropolitan cities were obtained from the database of census data and governmental surveys [34], and were used as regional socioeconomic variables.

### Statistical analysis

Multilevel analysis was performed with data for 20,003 men and 21,076 women (level-1) nested within 60 prefectures/metropolitan cities (level-2). To estimate the average relationships between health risk behaviours and individual variables across all regions (individual fixed parameters), the variations between prefectures/metropolitan cities that could not be accounted for by individual factors (regional random variance), and the effects of regional variables on health risk behaviours (regional fixed parameter), a multilevel binomial non-linear logit link model using the iterative generalised least-squares (IGLS) was fitted [35].

Individual and regional fixed parameters were expressed by the adjusted odds ratio (OR) with 95% confidence interval (CI). The proportion of variance related to the region (intra-regional correlation:%) was approximated as: regional variance/(regional variance + π^2^/3) ×100 [36,37]. Statistical analyses were performed using MLwiN version 1.10 [38].

## Results

Table 1 shows the basic characteristics of the subjects included in the present study. The mean age was 42.9 years for both men and women. A large proportion of the subjects were classified as "married": 73.4% for men and 76.9% for women. The majority of women reported their occupation as "housework" (27.4%). In the following analysis, "protective service" and "housework" for men, and "manager", "protective service," and "transport" for women were included in "others", because they each comprised only a very small proportion of the total. In multilevel analysis, the references were "profession" for men and "housework" for women. Per capita income and unemployment rate of 60 prefectures/metropolitan cities ranged from 865 to 2,042 (thousand yen) and 3.0 to 9.4 (%), respectively.

**Table 1:** Basic characteristics of study subjects and regions.

		Men	Women
*Individual variable *(20030 men and 21076 women)			
Age (mean, range: years)		42.9 (25–59)	42.9 (25–59)
Marital status (N, %)	Married	14701 (73.4)	16208 (76.9)
	Single	4635 (23.1)	3217 (15.3)
	Separated	137 (0.7)	541 (2.6)
	Divorced	557 (2.8)	1110 (5.3)
			
Occupation ^a ^(N, %)	Profession	3747 (18.7)	2243 (10.6)
	Manager	1736 (8.7)	310 (1.5)
	Clerk	1776 (8.9)	3051 (14.5)
	Sales work	1972 (9.8)	1785 (8.5)
	Service work	1697 (8.5)	2242 (10.6)
	Agriculture	680 (3.4)	535 (2.5)
	Transport	1063 (5.3)	80 (0.4)
	Labour	4752 (23.7)	2167 (10.3)
	Housework	28 (0.1)	5765 (27.4)
	Others	2579 (12.9)	2898 (13.8)
			
Income quintile (median: thousand yen)	5th (highest)	6035	
	4th	4000	
	3rd	2989	
	2nd	2200	
	1st (lowest)	1250	
*Regional variable *(60 prefectures and metropolitan cities)			
Per capita income (mean, range: thousand yen)		1423.4 (865–2042)	
Unemployment rate (mean, range: %)		4.68 (3.0–9.4)	

a Profession = professional and technical workers; clerk = clerical and related workers; agriculture = agriculture, foresty and fishery workers; labour = craftmen, mining, production process and construction workers, and labourers.

The prevalence of difference health risk behaviours are shown in Table 2. Current smoking, excessive alcohol consumption and poor dietary habits were much more prevalent in men (57.3, 27.4 and 40.6%, respectively) than in women (15.6, 5.8 and 27.4%, respectively). Physical inactivity and stress were not markedly different between men and women, but women showed a higher prevalence of non-attendance of health check-ups than men (42.3% *vs*. 27.5%, respectively).

**Table 2:** Prevalence of health risk behaviour in Japanese adults

Health risk behavior	Men		Women	
		
	N	(%)	N	(%)
Current smoking	10784	(57.3)	3099	(15.6)
Excess alcohol consumption	5177	(27.4)	1155	(5.8)
Physical inactivity	13989	(71.2)	14926	(72.0)
Poor dietary habits	7974	(40.6)	5671	(27.4)
Stress	9639	(51.9)	12151	(61.3)
Non-attendance of health check-ups	5243	(27.5)	8532	(42.3)

Table 3 shows the results of multilevel analysis of the relationships between health risk behaviours and individual and regional socioeconomic variables for men. Compared to married subjects, those who were divorced showed significantly higher OR for current smoking, poor dietary habits and non-attendance of health check-ups. Subjects in lower occupational classes, such as "service work", "transport," and "labour", showed significantly higher OR for risk behaviours as compared to those classified as "professional". There was a significant gradient of increased OR according to income, except with regard to excessive alcohol consumption and stress.

**Table 3:** Results of multilevel analysis for health risk behaviour in Japanese men: odds ratio (OR) and 95% confidence interval (95%CI) of individual and regional variables.

Variable	Current smoking (N = 19816)		Excess alcohol consumption (N = 18922)		Physical inactivity (N = 20728)		Poor dietary habits (N = 20728)		Stress (N = 19817)		Non-attendance of health check-up (N = 20148)	
						
Individual	OR	(95%CI)	OR	(95%CI)	OR	(95%CI)	OR	(95%CI)	OR	(95%CI)	OR	(95%CI)
Individual												
Age (10 years)	0.81	(0.78, 0.84) ***	1.17	(1.12, 1.21) ***	0.93	(0.90, 0.97) *	0.71	(0.69, 0.74) ***	1.01	(0.98, 1.04)	0.88	(0.84, 0.91) ***
Marital status												
Married	1.00		1.00		1.00		1.00		1.00		1.00	
Single	0.72	(0.66, 0.78) ***	0.62	(0.56, 0.68) ***	0.98	(0.90, 1.07)	1.04	(0.96, 1.13)	0.82	(0.75, 0.88) ***	1.73	(1.58, 1.89) ***
Widow	1.35	(0.93, 1.95)	0.83	(0.55, 1.25)	1.08	(0.72, 1.62)	1.28	(0.87, 1.87)	0.97	(0.67, 1.41)	1.11	(0.71, 1.74)
Divorced	1.89	(1.55, 2.31) ***	1.10	(0.91, 1.33)	1.14	(0.93, 1.40)	1.28	(1.06, 1.53) *	1.06	(0.88, 1.27)	1.75	(1.43, 2.12) ***
Occupation ^a^												
Profession	1.00		1.00		1.00		1.00		1.00		1.00	
Manager	1.14	(1.01, 1.28) *	1.07	(0.93, 1.22)	0.94	(0.83, 1.07)	0.92	(0.81, 1.05)	1.03	(0.92, 1.16)	0.78	(0.67, 0.92) ***
Clerk	0.94	(0.84, 1.06)	0.97	(0.85, 1.11)	1.06	(0.93, 1.20)	0.96	(0.85, 1.09)	0.99	(0.88, 1.11)	0.59	(0.50, 0.69) ***
Sales work	1.31	(1.17, 1.47) ***	1.18	(1.04, 1.34) *	1.11	(0.98, 1.25)	1.06	(0.94, 1.19)	1.01	(0.90, 1.13)	1.68	(1.47, 1.91) ***
Service work	1.26	(1.12, 1.42) ***	1.13	(0.98, 1.29)	1.20	(1.05, 1.37) *	1.19	(1.06, 1.35) **	1.07	(0.95, 1.21)	1.34	(1.17, 1.54) ***
Agriculture	1.16	(0.97, 1.39)	1.20	(0.99, 1.46)	1.03	(0.85, 1.25)	0.93	(0.77, 1.11)	0.73	(0.61, 0.87) ***	1.84	(1.52, 2.24) ***
Trasnport	1.61	(1.39, 1.87) ***	1.29	(1.10, 1.51) **	1.19	(1.01, 1.39) *	1.35	(1.16, 1.56) ***	0.92	(0.80, 1.06)	1.08	(0.91, 1.28)
Labour	1.49	(1.36, 1.63) ***	1.26	(1.14, 1.39) ***	1.13	(1.03, 1.25) *	1.12	(1.02, 1.23) *	0.89	(0.81, 0.98) *	1.10	(0.99, 1.22)
Others	1.08	(0.97, 1.20)	0.91	(0.80, 1.03)	0.90	(0.80, 1.01)	1.00	(0.89, 1.11)	1.03	(0.92, 1.15)	1.33	(1.17, 1.50) ***
Income quintile												
5th (highest)	1.00		1.00		1.00		1.00		1.00		1.00	
4th	1.11	(1.01, 1.21) *	0.96	(0.87, 1.06)	1.17	(1.06, 1.29) ***	1.08	(0.98, 1.19)	1.10	(1.00, 1.20) *	1.10	(0.98, 1.24)
3rd	1.12	(1.02, 1.23) *	0.99	(0.89, 1.10)	1.31	(1.19, 1.45) ***	1.16	(1.06, 1.28) **	1.07	(0.97, 1.17)	1.34	(1.19, 1.50) ***
2nd	1.30	(1.18, 1.43) ***	1.03	(0.92, 1.14)	1.43	(1.29, 1.58) ***	1.26	(1.14, 1.39) ***	1.09	(0.99, 1.20)	1.99	(1.77, 2.23) ***
1st (lowest)	1.29	(1.17, 1.43) ***	0.99	(0.89, 1.10)	1.42	(1.28, 1.58) ***	1.28	(1.16, 1.42) ***	1.15	(1.05, 1.27) **	3.14	(2.80, 3.52) ***
Region												
Per capita income (million yen)	0.89	(0.71, 1.11)	1.03	(0.82, 1.29)	1.05	(0.92, 1.20)	1.09	(0.94, 1.26)	1.44	(1.24, 1.67) ***	1.42	(1.15, 1.76) **
Unemployment (%)	1.06	(1.01, 1.12) *	1.07	(1.01, 1.13) *	0.99	(0.96, 1.02)	1.02	(0.99, 1.06)	1.01	(0.98, 1.05)	1.11	(1.05, 1.16) **
Regional random variance (SE) ^b^	0.029	(0.008) ***	0.028	(0.008) ***	0.000	(0.000)	0.004	(0.003)	0.005	(0.003)	0.029	(0.009) **
-2 log likelihood	26317.0		22089.1		23313.1		25487.2		26207.1		19463.1	
Intra-regional correlation (%)	8.0		7.7		0.0		1.1		1.6		8.0	

* p < 0.05, ** p < 0.01, *** p < 0.001

a Profession = professional and technical workers; manager = managers and officials; clerk = clerical and related workers; agriculture = agriculture, forestiry and fishery workers; transport = workers in transport and communications; labour = craftmen, mining, production process, and construction workers and labourers.

b Variance at the regional level in a logit model

With regard to regional indicators, significant correlations were found between per capita income and stress and non-attendance of health check-ups, and between unemployment rate and current smoking, excessive alcohol consumption and non-attendance of health check-ups. Significant regional variance was observed for current smoking, excessive alcohol consumption and non-attendance of health check-ups.

Table 4 shows the results of multilevel analysis for women. Being divorced showed a significantly higher OR for current smoking, excessive alcohol consumption, poor dietary habits and stress. Non-attendance of health check-ups showed significantly lower OR for all types of employment as compared to "housework", while no significant differences were observed between OR for other health risk behaviours and any type of employment except for the relationship between current smoking and "agriculture". Higher income was associated with reduced likelihood of all risk behaviours. The strongest relationships were found for current smoking and non-attendance of health check-ups, which showed approximately two-fold greater odds in the lowest quintile as compared to the highest quintile.

**Table 4:** Results of multilevel analysis for health risk behaviour in Japanese women: odds ratio (OR) and 95% confidence interval (95%CI) of individual and regional variables.

Variable	Current smoking (N = 19816)		Excess alcohol consumption (N = 18922)		Physical inactivity (N = 20728)		Poor dietary habits (N = 20728)		Stress (N = 19817)		Non-attendance of check-up (N = 20148)	
						
	OR	(95%CI)	OR	(95%CI)	OR	(95%CI)	OR	(95%CI)	OR	(95%CI)	OR	(95%CI)
Individual												
Age (10 years)	0.76	(0.73, 0.79) ***	0.93	(0.87, 1.00)	0.70	(0.65, 0.75)***	0.70	(0.68, 0.73) ***	0.93	(0.90, 0.96) **	0.65	(0.63, 0.68) ***
Marital status												
Married	1.00		1.00		1.00		1.00		1.00		1.00	
Single	1.00	(0.89, 1.14)	1.00	(0.82, 1.21)	0.73	(0.65, 0.81) **	1.12	(1.01, 1.24) *	0.73	(0.66, 0.80) ***	0.63	(0.57, 0.70) ***
Widow	1.07	(0.82, 1.40)	0.76	(0.49, 1.19)	1.11	(0.91, 1.35)	1.09	(0.87, 1.36)	0.92	(0.76, 1.11)	0.82	(0.67, 1.02)
Divorced	2.67	(2.30, 3.09) ***	1.68	(1.33, 2.11) ***	1.09	(0.93, 1.27)	1.23	(1.06, 1.42) **	1.16	(1.01, 1.34) *	0.88	(0.76, 1.02)
Occupation ^a^												
Housework	1.00		1.00		1.00		1.00		1.00		1.00	
Profession	1.08	(0.92, 1.25)	1.34	(1.06, 1.70) *	1.01	(0.90, 1.14)	1.03	(0.91, 1.17)	1.32	(1.18, 1.47) ***	0.26	(0.24, 0.30) ***
Clerk	0.97	(0.84, 1.12)	1.46	(1.19, 1.80) ***	1.15	(1.03, 1.28) **	1.06	(0.95, 1.19)	1.20	(1.09, 1.33) ***	0.28	(0.25, 0.31) ***
Sales work	1.57	(1.35, 1.82) ***	1.79	(1.42, 2.25) ***	1.11	(0.98, 1.25)	1.43	(1.26, 1.62) ***	1.19	(1.06, 1.34) **	0.48	(0.43, 0.54) ***
Service work	1.58	(1.37, 1.81) ***	1.91	(1.55, 2.36) ***	1.10	(0.98, 1.23)	1.37	(1.22, 1.54) ***	1.14	(1.03, 1.28) *	0.42	(0.37, 0.47) ***
Agriculture	0.70	(0.52, 0.96) *	1.21	(0.79, 1.86)	0.99	(0.81, 1.22)	1.06	(0.85, 1.32)	0.84	(0.70, 1.02)	0.56	(0.46, 0.68) ***
Labour	1.10	(0.94, 1.28)	1.07	(0.83, 1.38)	1.35	(1.20, 1.52) ***	1.38	(1.23, 1.56) ***	1.06	(0.95, 1.18)	0.36	(0.32, 0.40) ***
Others	1.30	(1.13, 1.48) ***	1.34	(1.08, 1.66) ***	1.03	(0.93, 1.15)	1.22	(1.09, 1.36) **	1.06	(0.96, 1.17)	0.53	(0.48, 0.59) ***
Income quintile												
5th (highest)	1.00		1.00		1.00		1.00		1.00		1.00	
4th	1.12	(0.97, 1.29)	0.96	(0.78, 1.17)	1.06	(0.96, 1.17)	1.18	(1.06, 1.32) **	1.06	(0.97, 1.17)	1.21	(1.09, 1.34) ***
3rd	1.34	(1.16, 1.54) **	1.04	(0.85, 1.27)	1.11	(1.00, 1.22) *	1.24	(1.11, 1.39) ***	1.11	(1.01, 1.22) *	1.42	(1.29, 1.58) ***
2nd	1.66	(1.44, 1.90) ***	1.06	(0.86, 1.29)	1.21	(1.09, 1.34) ***	1.32	(1.19, 1.48) ***	1.14	(1.04, 1.26) **	1.75	(1.58, 1.93) ***
1st (lowest)	2.03	(1.76, 2.33) ***	1.28	(1.04, 1.56) *	1.24	(1.12, 1.38) ***	1.64	(1.47, 1.83) ***	1.26	(1.14, 1.39) ***	2.23	(2.01, 2.47) ***
Region												
Per capita income (million yen)	1.93	(1.64, 2.28) **	1.49	(1.05, 2.10) *	0.76	(0.64, 0.90) **	1.12	(0.90, 1.40)	1.43	(1.23, 1.67) ***	1.75	(1.13, 2.27) ***
Unemployment (%)	1.08	(0.99, 1.18)	1.10	(1.03, 1.19) *	0.95	(0.92, 0.99) **	1.03	(0.98, 1.08)	1.00	(0.97, 1.04)	1.09	(1.03, 1.15) **
Regional random variance (SE) ^b^	0.118	(0.026) ***	0.043	(0.019) *	0.009	(0.004) *	0.032	(0.009) ***	0.007	(0.004)	0.045	(0.011) ***
-2 log likelihood	13564.2		-2382.9		23140.5		23489.1		26988.4		23946.7	
Intra-regional correlation (%)	26.1		11.4		2.6		8.8		2.1		11.8	

* p < 0.05, ** p < 0.01, *** p < 0.001

a Profession = professional and technical workers; clerk = clerical and related workers; agriculture = agriculture, foresty and fishery workers; labour = craftmen, mining, production process and construction workers, and labourers.

b Variance at the regional level in a logit model

Per capita income and unemployment rate showed significant positive associations with current smoking, excessive alcohol consumption, stress and non-attendance of health check-ups. With the exception of stress, all risk behaviours showed significant regional variance. All risk behaviours showed higher intra-regional correlations as compared to men: in particular, 26.1% for current smoking, 11.8% for non-attendance of health check-ups and 11.4% for excessive alcohol consumption.

## Discussion

The results of the present study indicated a substantial relationship between health risk behaviour and lower socioeconomic status at the level of the individual in Japanese men and women. The regional variance and the influence of regional socioeconomic indicators on risk behaviour were marked in women, but small in men.

Among men, those in the lower occupational classes showed a higher likelihood of risk behaviours, except for stress, as compared to "professionals". Especially, "service work," "transport" and "labour" showed significantly higher likelihood of current smoking, excessive alcohol consumption, physical inactivity and poor dietary habits. These observations suggest a plausible explanation for the higher mortality rates among these occupational classes noted in the national data [27].

Individual income was significantly related to risk behaviour of smoking, exercise, diet and health check-ups in both men and women, and lower income increased the likelihood of these behaviours. In men, a clear gradient of OR was found only for non-attendance of health check-ups and the gradient of OR for risk behaviours was not clearer than that of women. The model without occupation showed a clear gradient of OR in men [39] indicating a substantial degree of income-related inequality in health behaviour and its interaction with occupational class in Japanese men.

For women, no occupation showed a significantly lower OR as compared to "housework", with the exception of current smoking for "agriculture" and non-attendance of health check-ups for all occupational categories. This suggests that employment is associated with risk behaviour in women. Previous studies demonstrated that women's participation in society was related to a higher prevalence of smoking in accordance with the reduced intolerance toward this habit in women [40,41]. In addition, the tendency for higher OR of current smoking, excessive alcohol consumption and poor dietary habits in "sales workers" and "service workers" among the occupational categories implies the accumulation of risk behaviours in Japanese women in lower occupational classes.

It is interesting that excessive alcohol consumption did not show an income-related gradient in either men or women: the second highest quintile in men showed a significantly lower OR and the lowest quintile in women showed a significantly higher OR as compared to the highest quintile. For women, the difference in excessive alcohol consumption by occupation was greater than those in the other health risk behaviours. A previous study confirmed that participation in the workforce increases women's drinking habit in Japan [42].

The relationship between individual socioeconomic status and non-attendance of health check-ups showed a different pattern from other behaviours. Women in the "housework" category and men in the "agriculture" category were less likely to attend health check-ups. Health check-ups in people of working age are strongly dependent on the workplace [43]. Employees, particularly in large companies, have greater benefits of not only occupational health services but also preventive health services [44,45]. The steepest gradient of OR for non-attendance of health check-ups among all health risk behaviours suggests substantial inequality in receiving preventive health services according to socioeconomic status in the Japanese population.

There was a clear gender difference in the influence of regional socioeconomic indicators on health risk behaviour. All risk behaviours showed higher intra-regional correlations in women than in men, and marked differences were found for current smoking and excessive alcohol consumption. Area socioeconomic conditions have been shown to influence health-related behaviour, and in general those living in socioeconomically disadvantaged areas have a higher likelihood of health risk behaviour [7,13,23,46]. However, similarly to previous studies in France showing correlations between higher gross domestic product per capita in residential areas and both smoking and alcohol consumption [47,48], the results of the present study indicated that women living in areas with higher per capita income had higher likelihood of current smoking, excessive alcohol consumption, stress and non-attendance of health check-ups. This may be explained by the following two points.

First, the regional differences in socioeconomic conditions in Japan are relatively small, and thus, regional disadvantage and deprivation would have little influence on individual health-related behaviour in the Japanese population. The data indicated that the degree of income inequality in Japan is smaller than in other industrial countries [49,50], and the regional inequality in per capita income has decreased over the past several decades [51]. As a previous study demonstrated that national financial adjustment policy contributed to a reduction of regional disparity in health levels [52], a national minimum across the country was achieved by egalitarian social policies in Japan.

A second explanation is related to the linkage between per capita income and urban-rural differences. In Japan, indicators reflecting urbanisation, such as population size and population density, are strongly correlated with higher income, and therefore income-related indicators represent not only socioeconomic conditions but also aspects of urban-rural differences – higher per capita income indicates an urban context [28,53,54]. Therefore, the results of the present study imply a relationship between a higher likelihood of health risk behaviour and urbanisation. Urbanisation accompanied by social participation of women is likely to increase the likelihood of smoking and alcohol drinking in Japan [40-42].

One notable feature of the geographical variation in Japan is the deteriorating relative health levels of urban populations, especially for women. Osaka Prefecture, which is the second largest metropolis after the Tokyo Metropolitan Area, had the second shortest life expectancy for women among the 47 prefectures in Japan [55]. In addition, life expectancy in the Tokyo Metropolis, which had the longest life expectancy before 1965, ranked 15th for men and 37th for women in 2000 [55]. Urban areas showed higher mortality rates from cancer and ischemic heart disease than rural areas [56], and individual health-related behaviour contributes strongly to these diseases [23,57,58].

For men, higher mortality rates are found in areas with lower income- and education-related indicators [28]. As shown in the present study, regional socioeconomic indicators had little influence on health risk behaviour in men taking individual socioeconomic indicators into consideration. As mentioned above, a previous study indicated a gender-related difference in the relationship between mortality and area socioeconomic status: higher mortality rates in areas with lower per capita income were seen only in men [28]. The higher likelihood of health risk behaviour in men on the lower socioeconomic scale suggests one plausible explanation for the higher mortality in areas with lower per capita income, where lower-income individuals are more likely to live. In contrast, the relationship between health risk behaviour and higher per capita income can explain the marked deterioration of health level in women in urban areas with higher per capita income.

Finally, it is necessary to discuss the limitations of this study, as well as its strengths. The present study was performed in a large nationally representative sample with stratified random sampling, although potential differences in response rates based on socioeconomic status and region may have caused selection bias [59,60]. The questionnaire regarding health risk behaviour was comprehensive, although it was self-reported and a few behaviours (*e.g.*, poor dietary habits and physical inactivity) are quite subjective. The items of dietary habits were based on the national guidelines for a healthy diet [61], and they (or the index formulated using these items) have been shown to be related to some aspects of physical health status [62,63]. Nevertheless, the validity and reliability of these questions have not been examined in detail, and those of single-item questions about physical activity were verified but only moderately so [64,65]. These issues probably induced misclassification bias [59,60].

In this study, we effectively applied multilevel analysis to elucidate the influence of socioeconomic factors of two levels and to demonstrate regional variances. However, our models did not consider random effects of the variables or interactions between the variables. Supplemental analysis using random slope models for household income did not show statistically significant regional variance in the effects of household income on any health risk behaviour in either men or women (data not shown).

Other limitations are related to the measurements of socioeconomic status. In the present study, household income was used as the main measure of individual socioeconomic status, which was estimated from the details of income-related information and adjusted for family size and composition. Educational attainment is also commonly used as another major measure of socioeconomic status [66,67]. As the survey used in the present study did not include educational information, no education-related variable could be introduced into the analyses. Previous studies have shown that differences in health-related behaviour among groups stratified according to educational attainment in Japan are substantial [29,30,68]. Further studies are required to clarify the independent and interactive influences of different socioeconomic measures on health risk behaviour.

## Conclusion

The results of the present study demonstrated a close link between selected health risk behaviours and individual socioeconomic status in the Japanese population. A lower socioeconomic status measured according to income and occupation was generally associated with higher likelihood of health risk behaviours, such as current smoking and excessive alcohol consumption. Regional variance and the independent influence of regional indicators were marked in women, but small in men, and higher per capita income, reflecting an urban context, was related to accumulation of risk behaviours in women.

## Competing interests

The author(s) declare that they have no competing interests.

## Authors' contributions

YF designed the study, analyzed the data, and drafted the article. KN helped to interpret the results and edited the draft. TT supervised the data analysis and writing article.

## Pre-publication history

The pre-publication history for this paper can be accessed here:


